# Continuous renal replacement therapy with the adsorbent membrane oXiris in septic patients: a clinical experience

**DOI:** 10.1186/cc12001

**Published:** 2013-03-19

**Authors:** F Turani, F Candidi, R Barchetta, E Grilli, A Belli, E Papi, A di Marzio, M Falco

**Affiliations:** 1 Aurelia Hospital/European Hospital, Rome, Italy

## Introduction

Renal failure is an important complication of sepsis and CRRT with adsorbing membranes may be useful in this clinical setting [1]. The aims of the study in septic/septic shock patients are to evaluate: the safety of a new hemofilter membrane oXiris with adsorbing properties and anti-endotoxin activity; the renal and hemodynamic response; and the changes of endotoxin and proinflammatory molecules.

## Methods

Forty septic/septic shock patients with renal failure were enrolled in the study. All patients had preoperative endotoxin >0.6 level/units (EAA Spectral D) and were submitted to high-volume hemodiafiltration (50 ml/kg/hour, Prismaflex; Gambro) with a new treated heparin-coated membrane (oXiris; Gambro). At T0 (pre-treatment) and T1 (24 hours) the main clinical and biochemical data were evaluated. All data are expressed as mean ± SD. One-way ANOVA test with Bonferroni correction was used to evaluate the data changes. *P *< 0.05 was considered significant.

## Results

Table 1 presents the main results of this study.

**Table 1:** 

Parameter	Units	T0	T1
Creatinine	mg/dl	1.9 ± 0.1	1.18 ± 0.1*
Diuresis	ml/24 hours	1,284 ± 78	1,573 ± 98
Norepinephrine	μg/kg/minute	0.17 ± 0.2	0.06 ± 0.1*
IL-6	pg/ml	572 ± 78	278 ± 57*
Procalcitonin	ng/ml	35 ± 7	15 ± 2*
Endotoxin	Level/U	0.64 ± 0.2	0.49 ± 0.1

*P <0.05 vs. T0.

## Conclusion

In septic/septic shock patients with renal failure, CRRT with a new treated heparin-coated membrane (oXiris; Gambro) is clinically feasible, and has a positive effect on renal function and hemodynamics. An adsorbing effect on proinflammatory mediators may have a role in these results. These data and the trend toward a decrease of endotoxin during the treatment warrant further investigation.
